# Piloting a livestock identification and traceability system in the northern Tanzania–Narok–Nairobi trade route

**DOI:** 10.1007/s11250-017-1431-4

**Published:** 2017-09-30

**Authors:** Florence Mutua, Absolomon Kihara, Jason Rogena, Nicholas Ngwili, Gabriel Aboge, James Wabacha, Bernard Bett

**Affiliations:** 1 International Livestock Research Institute, Nairobi, Kenya; 2 Department of Public Health, Pharmacology & Toxicology, Faculty of Veterinary Medicine, University of Nairobi, Nairobi, Kenya; 3 African Union Inter-African Bureau for Animal Resources, Nairobi, Kenya

**Keywords:** Livestock identification and traceability, Animal health and food safety surveillance

## Abstract

We designed and piloted a livestock identification and traceability system (LITS) along the Northern Tanzania–Narok–Nairobi beef value chain. Animals were randomly selected and identified at the primary markets using uniquely coded ear tags. Data on identification, ownership, source (village), and the site of recruitment (primary market) were collected and posted to an online database. Similar data were collected in all the markets where tagged animals passed through until they got to defined slaughterhouses. Meat samples were collected during slaughter and later analyzed for tetracycline and diminazene residues using high-performance liquid chromatography (HPLC). Follow up surveys were done to assess the pilot system. The database captured a total of 4260 records from 741 cattle. Cattle recruited in the primary markets in Narok (*n* = 1698) either came from farms (43.8%), local markets (37.7%), or from markets in Tanzania (18.5%). Soit Sambu market was the main source of animals entering the market from Tanzania (54%; *n* = 370). Most tagged cattle (72%, *n* = 197) were slaughtered at the *Ewaso Ng’iro* slaughterhouse in Narok. Lesions observed (5%; *n* = 192) were related to either hydatidosis or fascioliasis. The mean diminazene aceturate residue level was 320.78 ± 193.48 ppb. We used the traceability system to identify sources of animals with observable high drug residue levels in tissues. Based on the findings from this study, we discuss opportunities for LITS—as a tool for surveillance for both animal health and food safety, and outline challenges of its deployment in a local beef value chain—such as limited incentives for uptake.

## Introduction

Livestock identification and traceability systems (LITS) are increasingly being used to support animal production, trade, and public health interventions throughout the world. Animal identification is the use of unique identifiers and registration systems to identify animals individually or collectively by their epidemiologic units, while livestock traceability refers to ability to follow an animal or group of animals during all stages of its life (OIE 2017). Multiple livestock identification methods including ear notching, hot iron branding, and conventional ear tags have been used over time but these have evolved to electronic identification systems such as the radio-frequency identification (RFID) boluses and RFID ear tags (Bowling et al. 2008; Moreki et al. 2012). In Africa, LITS have been used successfully in a few countries (Botswana, South Africa, and Namibia) which export chilled and frozen beef to the European Union under an Economic Partnership Agreement. Botswana probably has a more advanced individual animal identification systems in the region. Namibia has a more or less system that is based on ear tag identification methods (Paskin 2004).

LITS are least developed in other African countries; traditional identification methods such as ear notching and branding that are not amenable for use in traceability are mainly used. These traditional marks can be specific for a given community or family largely at family levels, and often get progressively differentiated as animals are exchanged between communities or from one generation to the next (Landais 2001). Recent animal identification technologies including the use of RFID’s have been piloted in some areas in Kenya, Uganda, and South Sudan, mainly to curb livestock theft (Ekuam 2008). These approaches are largely uncoordinated and have only been applied in isolated areas.

The contribution of animal identification to disease surveillance and food safety can only be realized if identification initiatives are linked to traceability. Traceability systems can allow animals or their products to be followed through market channels back to farms of origin. Interventions can then be targeted to areas that are identified to be the source of health risks, be they pathogens or chemical hazards such as drug residues. The usefulness of livestock traceability in risk assessment is also critical (Caporale et al. 2001). The tools are becoming more important as consumers demand safe and wholesome animal source foods especially in the prime markets. This requires the use of marks that are unique, permanent, tamper proof, and linked to an official registration system (COMESA 2009).

Though most developing countries are eager to implement LITS so as to access lucrative international livestock markets (Marumo and Monkhei 2009), most production systems in the region do not have sufficient capacities to attain the required standards. Guidelines have been developed to guide the design and implementation of this measure (ICPALD 2015), but there are capacity challenges that have not been fully characterized. This study was implemented in a pastoral production system that supplied the northern Tanzania–Narok–Nairobi beef value chain, to identify opportunities for LITS in a production system that supplied local markets in Kenya. Meat and blood samples were collected from slaughtered animals and were used as outcomes for assessing reliability of the online trace-back system developed for the project.

## Methodology

The study was implemented between September 2014 and December 2015. A study design workshop was held at the start to define the scope of LITS to pilot and identify a value chain to use. The scope was defined by determining the expected precision, breadth, and depth of the system depending on the range of data that were to be collected. The system was considered as providing high-precision data as the unit of sampling was an animal rather than groups of animals. Breadth represented the range of data to be collected which included identification numbers and other animal characteristics such sex, color, age, owner, origin, and expected destination while depth defined points of commencement (primary markets) and termination of LITS (abattoirs). This implied that animals captured at abattoirs could reliably be traced back to the primary market, and where possible, the village of origin. Ear tags were identified as the key identification device to use but other livestock identification methods such as skin painting, back tags, and reticular boluses could be allowed.

### Description of livestock market routes

**Fig. 1:**
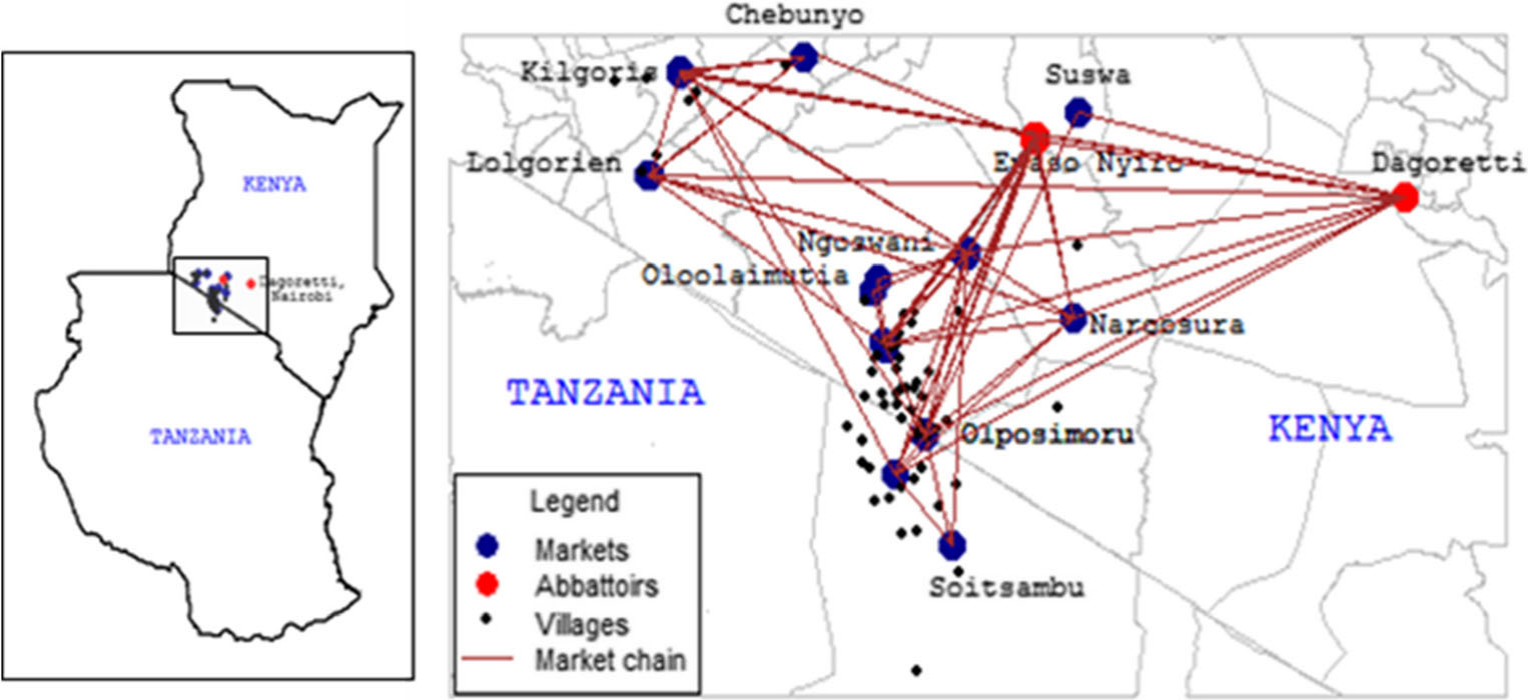
A map showing livestock markets and villages used for the LITS pilot study in Kenya and Tanzania

The Northern Tanzania–Narok–Nairobi beef value chain was mapped out during the study design workshop to determine existing primary and secondary livestock markets, trade routes, and slaughterhouses (Table 1; Fig. 1). Markets considered in the study included (1) primary markets located in the vicinity of Maasai Mara reserve (Olposimoru, Oloolaimutia, Lolgorian, Kilgoris, Soit Sambu), (2) secondary markets (Narosura, Ngoosuani, Ewaso Ng’iro, Chebunyo, Mulot, Ntulele, and Suswa), and (3) abattoirs (Ewaso Ng’iro, and Dagoretti). Activities implemented during the preparatory phase of the project included building new or repairing the existing crushes in the markets, training enumerators, and sensitizing market actors about the project.

**Table 1:** Livestock market routes in border areas of Narok (Kenya) and Ngorongoro (Tanzania)

Main routes considered in the pilot study	1. Soit Sambu (Tanzania)–Olposimoru–Narosura–Ngoosuani–Ewaso Ng’iro–Ntulele–Suswa–Dagoretti slaughterhouse
	2. Lolgorian–Kilgoris–Chebunyo–Mulot–Dagoretti
Additional routes identified during the follow up visits	3. Oloolaimutia–Sekenani–Ngoosuani–Ewaso Ng’iro slaughter, Goringori, Itong
	4. Soit Sambu–Olposimoru–Naikara (mainly shoats on Fridays)–Ngoosuani–Ewaso Ng’iro–Ewaso Ng’iro slaughterhouse
	5. Olmiti (in Tanzania), Endasikira, Muricho (loita)–Narosura–Ewaso Ng’iro–Ewaso Ng’iro slaughterhouse
	6. Ogwedhi–Kilogoris–Chebunyo–Mulot slaughterhouse, but also Kenya Meat Comission, Dagoretti slaughterhouse
	7. Olomesutie–Ilkerin–Narosura–Ewaso Ng’iro

### Development of an online data collection system

We designed electronic forms to capture data whenever tagged animals entered or exited study markets or upon entry into defined slaughterhouses using the ODK tool. These forms were subsequently uploaded into smart phones for data collection. Completed forms were, at the end of each day, uploaded to an online database system hosted at the ILRI server. The system was first pre-tested before being applied in the field. Cattle identified at the primary markets in Tanzania (mainly Soit Sambu market) were expected to enter the Narok market chain and would then be re-captured in the secondary markets in Kenya. There was also the possibility that animals identified in Kenya would enter markets in Tanzania through the same route. The system required defining a unique numbering system that captured identification details in both countries.

### Animal identification and collection of LITS data

Cattle were identified at the primary markets. The identification code used included unique combination of numbers representing country (Kenya or Tanzania), names of district, market, and individual animal numbers (1 = 1000) (Table 2). The identification, registration and traceability procedures followed are summarized in Annex 1. Traceability data, including animal identification number (ID), source, owner, destination, etc., were collected as animals moved from one market to another until arrival either at the Narok’s *Ewaso Ng’iro* or Nairobi’s Dagoretti abattoirs (Table 3). Post mortem observations were recorded and posted to the database using an abattoir form which ensured linkage of the findings to animal identification numbers.

**Table 2:** Numbering system used in the LITS pilot study

Country	Name of district	Name of primary market	Unique identification
Tanzania	Ngorongoro	Soit Sambu	TNGRS—0000
Kenya	Narok	Olposimoru	KNRKO—0000
Kenya	Narok	Oloolaimutia	KNRKT—0000
Kenya	Narok	Kilgoris	KNRKK—0000
Kenya	Narok	Lolgorian	KNRKL—0000

**Table 3:** Types of data collected for the LITS pilot study

Subject	Type of data collected
Market	Name
	GPS location
Animal	Identification number and method (ear tag, back tag, paint, bolus) used
	Source (farm or another market); for farm, the specific village name is provided
	Next destination (market, back to farm, slaughter)
	Sex
	Color
	Age category
	Entering or exiting the market
Owner (producer or trader)	Name
	Telephone contact
	Specify if entry or exit or stock check
Slaughterhouse	Traders details (name and phone contacts)
	Animal id
	Immediate source of the animal being presented
	Meat inspection result in summary
	Specify if sample is taken (barcode id of the sample linked to the animal id)
Laboratory	Sample identification number
	Tests done (antibiotic residue; brucellosis)

### Drug residue analyses

#### Sample collection

Liver, spleen, muscle, and blood samples were collected from tagged animals entering the slaughterhouses and used later to determine levels of tetracycline and diminazene aceturate drug residues. These samples were collected by meat inspectors based in each slaughterhouse. Samples were transported to the Public Health, Pharmacology and Toxicology laboratories in the Faculty of Veterinary Medicine, University of Nairobi, where they were stored at −20 °C until analyzed for the residues.

#### Laboratory analysis

Standards containing 0.001, 0.01, and 0.1 ng/ml of diminazene aceturate were prepared from stock solution of the trypanocidal drug. Similar standard solutions of tetracyclines with concentrations of 0.001, 0.01, and 0.1 ng/ml of the drug were also prepared. A mixture of muscle tissues was homogenized for 5 min and the homogenate centrifuged at 3000 rpm for 15 min. The supernatants were retained and filtered through 0.45-μm Nylon filter. Diminazene residues were extracted via octadecylsilane solid-phase extraction (SPE) tubes and cleaned with 10% methanol. Elusion was done with 90/10 0.025 M acetonitrile 1-octanesulphonic acid sodium salt with 2% glacial acetic acid in water. The elute was vacuum-dried and re-dissolved in mobile phase (30/70 acetonitrile/0.005 M octanesulphonic acid sodium salt in water containing 0.1% 9amine, pH 3.2—adjusted with glacial acetic acid). The sample was filtered through a 0.45-μm fiber syringe filter and then injected in to the HPLC auto-sampler. One gram of meat was homogenized in a blender for 2 min and then citric acid was added. The sample mixture was later treated with nitric acid, methanol, and deionized water. The suspension was vortexed and sonicated in an ultrasonic bath for 15 min. The sonicated samples were centrifuged, filtered through 0.45-μm Nylon filter, and then injected into the HPLC system for analysis.

### Questionnaire surveys

A questionnaire survey was implemented at the end of the pilot study to obtain perceptions on identification devices used, costs, and levels of acceptance of the LITS system in general. Traders, producers, middlemen, and transporters were interviewed.

### Data entry and analyses

Data posted onto the online database were retrieved and analyzed using Stata® Version 12.1. Descriptive analyses were done and resulting frequencies summarized using tables, graphs, and means. The software was also used to generate relationships that allowed for desired linkages to be made, for example, linking laboratory and post mortem data with animal identification and source details.

### Ethical clearance

The ethical approval for this work was obtained from ILRI’s Institutional Research Ethics Committee (IREC). All those who participated, mainly farmers, traders, and market leaders, had to provide an informed (verbal) consent before being engaged in the study.

## Results

Results are presented in three sections: (i) descriptive statistics on the LITS database, (ii) laboratory findings on drug residues and how LITS enables the identification of areas where cattle with high drug residue levels were sourced from, and (iii) results from questionnaire surveys used to obtain feedback on the LITS system.

### Analysis of the LITS database

#### Identification devices

Ear tags were the main devices used to identify animals in the study. A small sample of tagged cattle (*n* = 76 animals) was identified using paint. Back tags were also used to identify 37 animals; however, 24.3% (9/37) of these were removed as soon as they were put.

#### Records from the LITS database

The database captured a total of 4260 records either as entries (*n* = 2610) or exits (*n* = 1650) from the markets. Table 4 provides a summary of the actual number of animals entering and exiting each market in the study. Most records were captured during entry into the markets (61.2%; 2610/4260).

**Table 4:** Number of records captured during entry into and exit from selected primary and secondary markets, between October 2014 and December 2014

	Number of records captured		Total number of records
	Entry to market	Exit from the market	
Soit Sambu (Tanzania)	198	86	284
Olposimoru	728	504	1232
Chebunyo*	28	34	62
Ewaso Ng’iro*	60	48	108
Kilgoris	88	42	130
Naroosura	106	88	194
Ngoosuani	518	310	828
Oloolaimutia	604	354	958
Lolgorian	280	182	462
Suswa	0	2	2

*Market activities start early with possibilities of missing opportunities to capture details related to tagged animals entering or leaving the markets

#### Details of tagged animals entering the markets

**Fig. 2:**
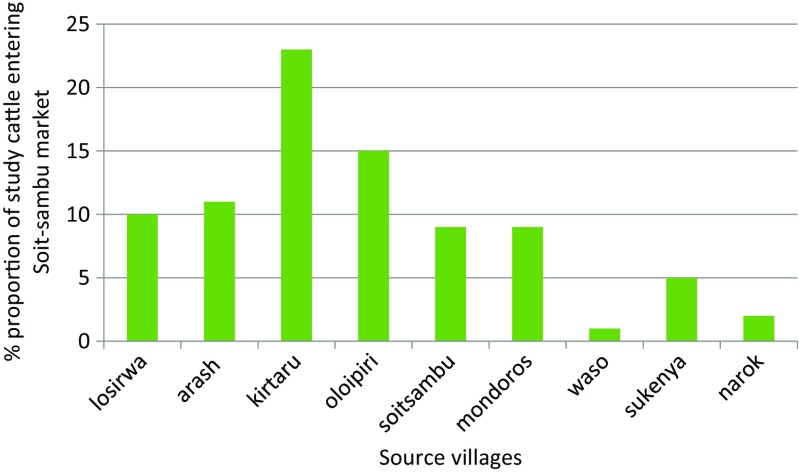
Sources of cattle marketed at the Soit Sambu livestock market in Ngorongoro, Tanzania

Cattle delivered for sale at the Tanzania’s Soit Sambu market had either been sourced from nearby farms (92%; 182/198) or were from local markets (8%; 16/198). Kirtalo (24%) and Oloipiri (16%) were the main source villages for animals sold at this border market of Tanzania (*n* = 182) (Fig. 2). Source markets (*n* = 16) included Karkarimoru (13%), Soit Sambu (50%), Piaya (6%), and the nearby Wasso (31%).

**Fig. 3:**
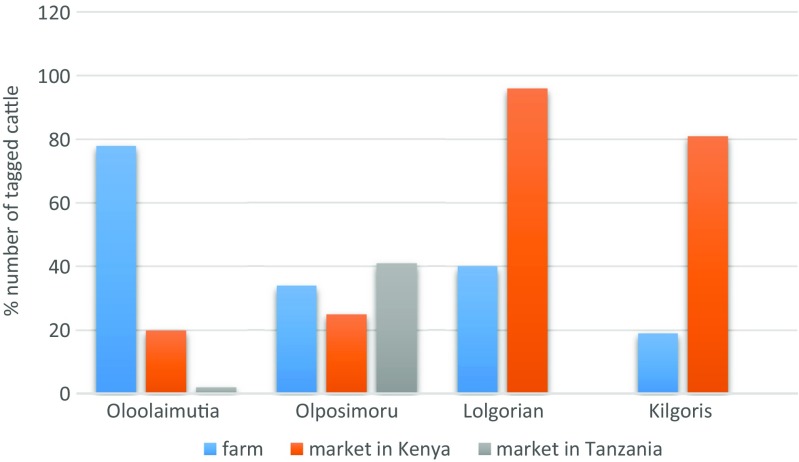
Sources of tagged cattle entering selected primary markets in the LITS pilot study

Sources of cattle sold at the selected primary markets in Kenya (*n* = 1698) included farms (43.8%), local markets (37.7%), and markets in Tanzania (18.5%). There were however variations across markets, for example, while most (78%; *n* = 604) cattle sold at the Oloolaimutia market had been sourced from farms, very few (19%; *n* = 86) of those sold at the Kilgoris market came from farms (Fig. 3). Soit Sambu was the main (54%; *n* = 370) border market in Tanzania supplying animals in the Narok–Nairobi livestock trade route. Other source markets included Kwitebe (1%) and Lolosukwa (13%).

#### Details of tagged animals exiting study markets

**Fig. 4:**
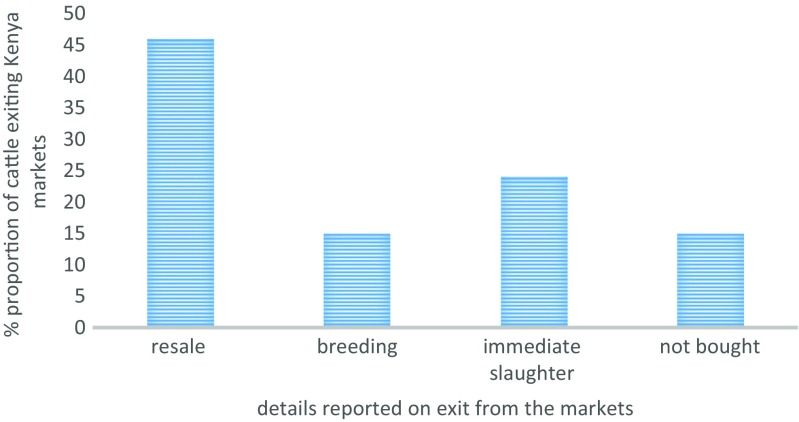
Details of animals exiting selected livestock markets in the LITS pilot study

Cattle exiting (*n* = 85) the Soit Sambu market in Tanzania had been bought by farmers wishing to keep them for breeding (17%), or purchased by traders wishing to sell in other markets (53%), or had not been sold out and were being taken back to the farms (26%). Cattle exiting the Kenyan markets (*n* = 1558) were mainly destined for sale in the next market level (46%) (Fig. 4).

Most cattle leaving the Ngoosuani (60%; *n* = 310) and Kilgoris (50%; *n* = 40) markets were for immediate slaughter (Table 5). Forty one percent (*n* = 182) of tagged cattle exiting the Lolgorian market had been bought for breeding.

**Table 5:** Details of animals exiting specific study markets

	Details of tagged animals exiting markets included in the LITS pilot study			
	% bought for breeding	% not sold	% to be traded in subsequent markets	% to be slaughtered
Lolgorian (*n* = 182)	41	19	35	5
Ngoosuani (*n* = 310)	12	5	23	60
Oloolaimutia (*n* = 354)	12	18	64	6
Olposimoru (*n* = 504)	10	20	60	10
Kilgoris (*n* = 40)	13	29	8	50

A small percentage of cattle leaving the Lolgorian market were to be traded in the border markets of Kihanja (6%), Mapera (3%), and Ntimaru (12%). Of the 302 exiting the Olposimoru market for sale in subsequent markets, 74% were destined for sale at the Ngoosuani market. Seventy seven percent (*n* = 62) of those exiting the Ngoosuani market were to be sold at the Ewaso Ng’iro market. Ewaso Ng’iro, Dagoretti, Njiru, and Kiserian were the main slaughterhouses where tagged animals were to be slaughtered (Table 6). Of the 24 slaughter animals exiting the Ewaso Ng’iro market, 66% were to be slaughtered at the nearby Ewaso Ng’iro slaughterhouse, 8% at the Narok slaughterhouse, and 26% in smaller slaughterhouses outside Narok.

**Table 6:** Number (%) of tagged animals exiting different markets and destined for slaughter in the different slaughterhouses

	Name of the slaughterhouse					
	Ewaso Ng’iro (%)	Dagoretti (%)	Kiserian/Ongata Rongai (%)	Njiru (%)	Narok (%)	Other slaughterhouses (%)
Ewaso Ng’iro (*n* = 24)	66	0	0	0	8	26
Chebunyo (*n* = 12)	0	17	17	33	0	33
Olposimoru (*n* = 54)	15	11	33	0	0	41
Ngoosuani (*n* = 186)	87	4	2	0	0	7
Lolgorian (*n* = 10)	0	40	0	0	0	60
Oloolaimutia (*n* = 22)	27	73	0	0	0	0

#### Records of tagged animals delivered to local slaughterhouses

**Fig. 5:**
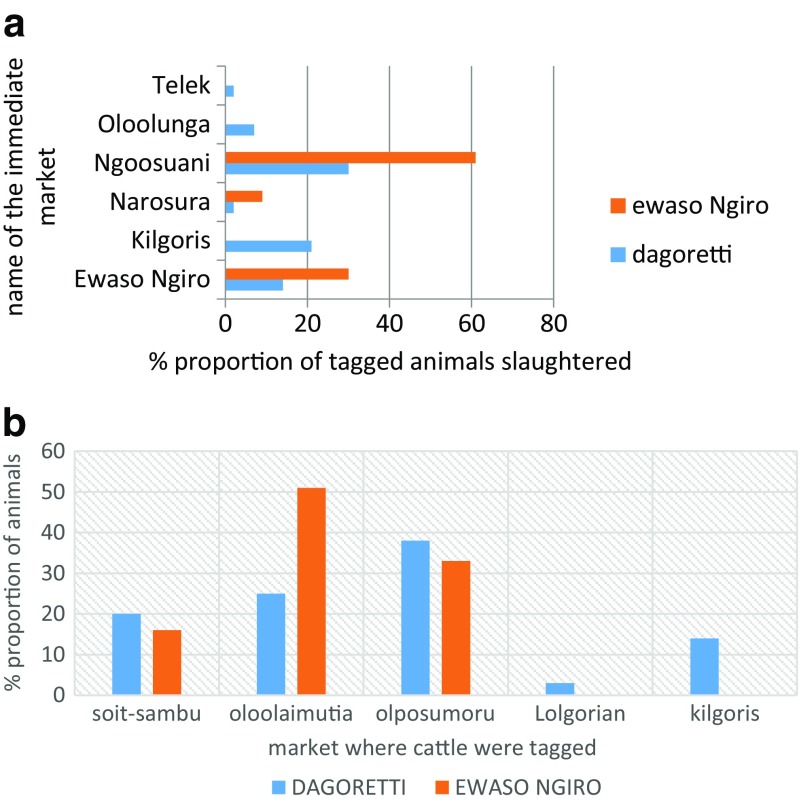
**a** Immediate source markets of tagged cattle slaughtered at the Dagoretti and the Ewaso Ng’iro slaughterhouses. **b** Primary sources of tagged cattle slaughtered at the Dagoretti and Ewaso Ng’iro slaughterhouses

A total of 197 tagged animals were received at the Ewaso Ng’iro (72%) and Dagoretti (28%) slaughterhouses. Ngoosuani market was immediate source market for cattle received both at Dagoretti slaughterhouse (53%; 30/56) and at the Ewaso Ng’iro slaughterhouse (60%; 86/141) (Fig. 5a). Cattle identified at the Soit Sambu market in Tanzania constituted 19.6% (11/56) and 15.6% (22/141) of animals slaughtered at the Dagoretti and Ewaso Ng’iro slaughterhouses, respectively (Fig. 5b).

#### Meat inspection results

Five out of the 197 tagged animals presented at the slaughterhouse were not slaughtered; three were sold on arrival at the Dagoretti slaughterhouse, to farmers wishing to fatten them while the rest were moved to a different slaughterhouse. The total number of tagged animals slaughtered was thus reduced to 192. The carcasses (95%, *n* = 192) were considered normal and were released for human consumption. Lesions (5%; *n* = 192) observed were related to either hydatidosis or fascioliasis.

### Drug residue analyses on meat samples

Where practically possible, multiple samples were collected from each animal; however, analyses were only done on 345 samples, mainly the liver and muscle tissues. Table 7 summarizes the type and number of samples taken from each slaughterhouse.

**Table 7:** Number of samples collected from tagged animals in the LITS pilot study

	Name of the slaughterhouse		
	Dagoretti (Nairobi)	Ewaso Ng’iro (Narok)	Total number of samples
Muscle	50	128	178
Liver	49	121	170
Kidney	51	80	131
Spleen	48	118	166
Blood	0	10	10

Three samples were discarded for errors (e.g., invisible labels). Diminazene aceturate was detected in 29 meat samples out of the 345 samples analyzed. The positive samples had diminazene aceturate residue levels ranging from 17.407 to 685.89 ppb. The recommended MRL for diminazene residues in meat updated in 2011 at the 34th Session of the Codex Alimentarius Commission is 500 ppb. Therefore, based on this MRL, 6 meat samples contained diminazene residues above the set MRL while the remaining 23 samples had trypanocidal residue levels below the MRL. Mean diminazene residue level was 320.78 ± 193.48 ppb (in samples with detectable amounts). None of the 345 samples analyzed tested positive for tetracycline residues.

### Perceptions on the piloted LITS system

Those interviewed (traders, brokers, and trekkers) had been in the livestock business for 8.7 ± 7.3 years and visited multiple markets each week. Slaughter animals were either trekked (94%) or were transported using trucks (6%). The animals were mostly moved during the day (92%). The mean number moved together was 29 ± 22 if trekked and 31 ± 26 if trucks were used. Those interviewed had their animals slaughtered either at the Ewaso Ng’iro (56%) or Dagoretti (44%) slaughterhouses. Slaughtering was done either during the day (21/52) or at night (34/52).

**Fig. 6:**
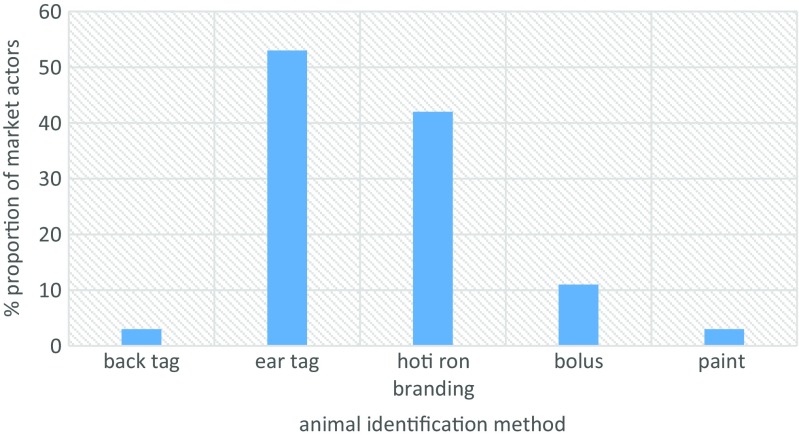
Preferred methods of animal identification

**Fig. 7:**
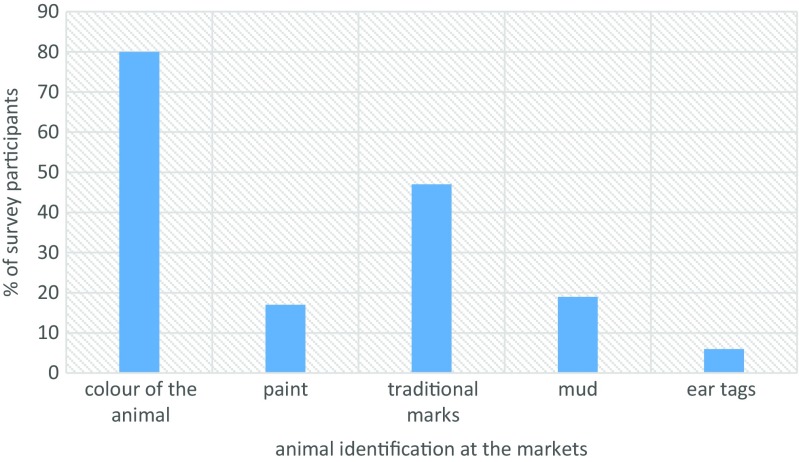
Animal identification methods used by stakeholders in livestock markets

**Fig. 8:**
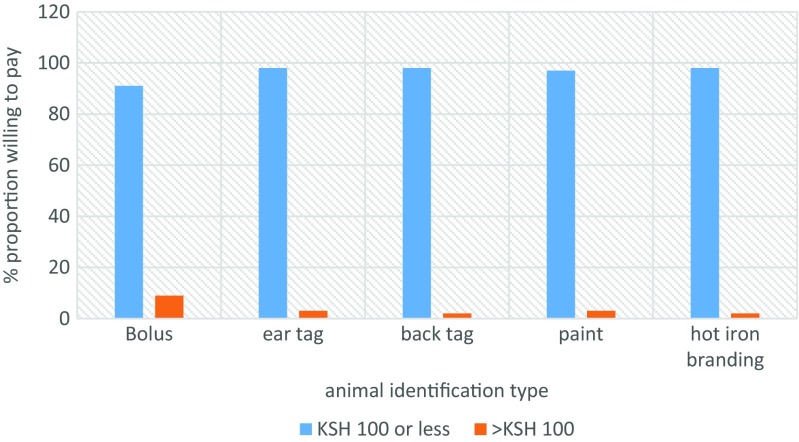
Perceived costs of various identification methods

**Fig. 9:**
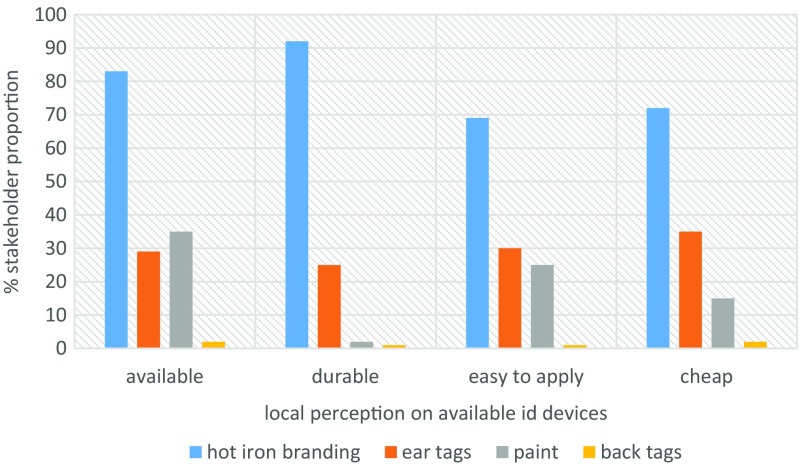
Perceptions on the use of hot-iron branding, ear tags, paint, and back tags

Ear tagging (53%) and hot iron branding (42%) were the most preferred methods of animal identification (Fig. 6). While at the markets, traders identified their animals visually using colors (80%) and brand marks (47%) (Fig. 7). We asked market actors to state how much, per animal, they would be willing to pay for if their animals were to be identified. Interestingly, for almost all device categories considered, stakeholder were willing pay ksh 100 (≈ USD 1) or less (Fig. 8). The traditional hot iron branding method was thought to be available (83%), durable (92%), easy to apply (69%), and affordable (72%) (Fig. 9).

Some of the traders feared trekking tagged animals as these could easily be confiscated by authorities particularly when crossing national borders in search of pasture. Traders disliked the use of paint as this would cause confusion in the market chain; cattle with paint marks are considered as already “sold” out. The traders were not conversant with the use of the bolus system but preferred using devises that are visible. Back tags were tampered with immediately they were applied. Traders were confident that none of those who bought breeding animals would attempt to remove ear tags. Some of the traders however preferred use of smaller ear tags.

### Using the database system in food safety surveillance

We used the database to (1) link positive samples from drug residue analysis to individual animal data (ear tag number, color of the animal, age, sex), (2) identify abattoirs where the animals had been slaughtered, and (3) determine other markets where the animal had passed through before arrival at the specified slaughterhouses. As an example, sample coded as TMP008113 tested positive for diminazene aceturate (874.59 ppb) and represented a black adult female identified as KNRKTO225. The animal, based on the tag number, had been sourced from Kenya, at the Oloolaimutia market, was traded through the Ngoosuani market before being slaughtered at the Ewaso Ng’iro slaughterhouse. A few other case details are summarized in Table 8.

**Table 8:**
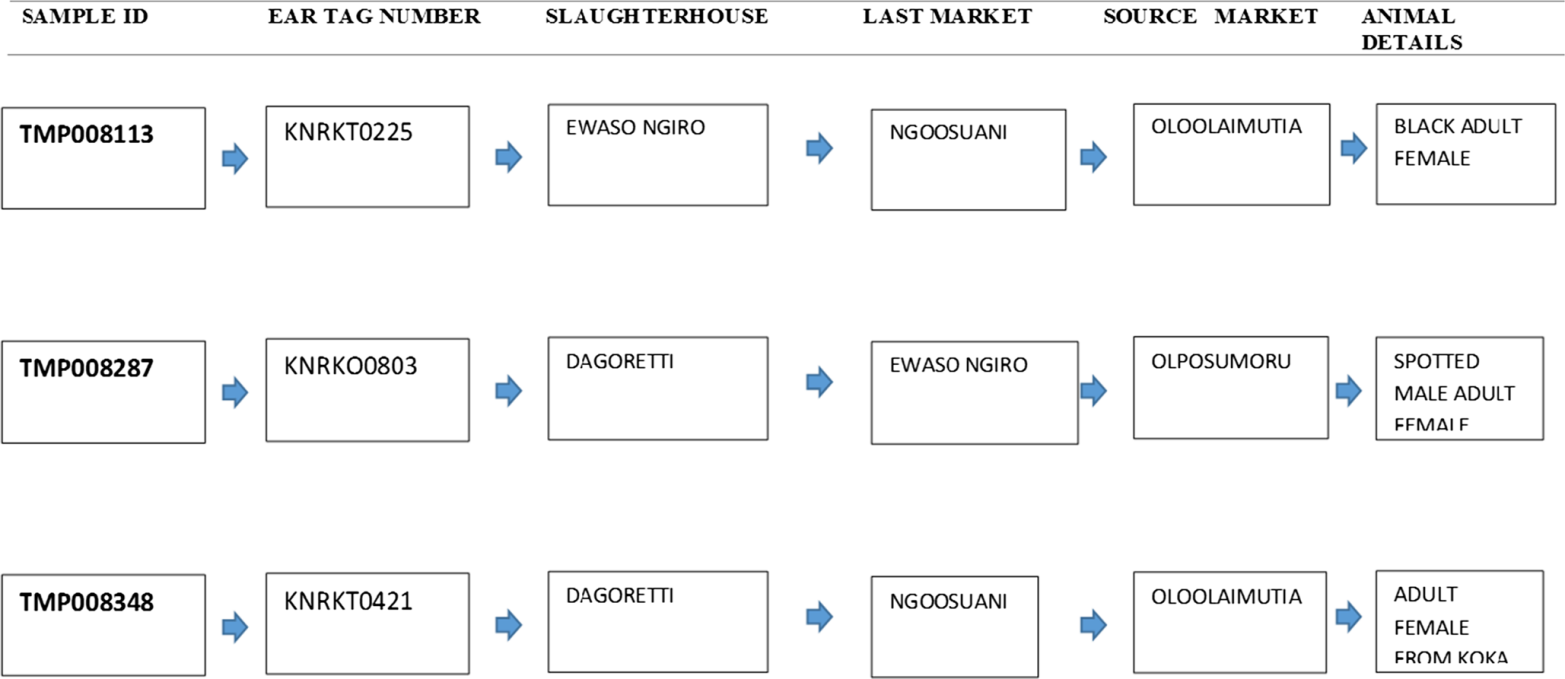
Traceability details for some of the samples with traceable levels (ppb) of diminazene aceturate residues

### Field experiences in LITS field implementation

Challenges encountered were related to either bad weather, data collection devices or were market-specific (Table 9).

**Table 9:** Summary of the challenges encountered in the field implementation of pilot animal identification and traceability along the northern Tanzania–Narok–Nairobi trade route

Challenge type	Market affected	Description of the challenge
Bad weather—wind, rain, sun	Narosura, Ngoosuani, and Ewaso Ng’iro	The roads became impassable when it rained; also interfered with data capture
Market-specific challenges	Chebunyo, Ewaso Ng’iro, Soit Sambu, Ngoosuani	▪ Some markets started very early with possibilities of missing out on tagged animals; some transactions being done outside the market yards—to evade taxes; weak and emaciated animals being offloaded outside the yards
Device and data system	–	▪ Most back tags were physically removed by the traders; Use of paint was not acceptable in some of the markets; Battery problems for both GPS and smart phones

## Discussion

The traceability system implemented in the study allowed for registration of an animal at the primary market and subsequent monitoring on other markets until the animal reached predetermined abattoirs in Narok or Nairobi. Although animals were tagged at the primary market, only a small percentage was traceable to source villages, presenting an opportunity to improve on the depth of the traceability system (i.e., tracing more animals to the village level from the primary markets). A desirable traceability system should also trace an animal to the farm level. However, in a pastoralist system where households are often organized into grazing units and animals get exchanged frequently between herds, a LIT system that terminates at the group level such as a village would suffice. Such a system would also enable an effective disease surveillance given that animals mix and come into direct contact, and might share a large proportion of infectious diseases, at the village or grazing unit levels.

One of the main challenges encountered in the study was obtaining accurate information on animal source. This is because most sellers encountered in the markets were middle men who had been used by producers to broker livestock sales. This practice has a detrimental effect of limiting retrieval of information on the source as well as the health of the animals being traded. This supports the clamor for the tagging of the animals at production units for completeness of traceability system, but in areas that have never had LITS before, a phased introduction of the measure, in line with the Pretoria declarations on LITS, would engender more fruitful outcomes.

Laboratory analyses revealed presence of high levels of diminazene aceturate in samples analyzed. The product is used a lot in management of trypanosomiasis in pastoralist areas and potentially presents a risk to development of resistance to related products in humans. This study did not detect any tetracycline residue in samples analyzed. The use of veterinary products without proper supervision by qualified animal health professionals is common in pastoralist areas of Kenya (Roderick et al. 2000). In such circumstances, LITS can then be used in conjunction with abattoir sampling to target areas where misuse of veterinary drugs is rampant. LITS can also allow for determination of sources of animals with important lesions and parasites observed during meat inspection. Interventions—such as information dissemination and education—can then be targeted to such areas to improve drug use practices. It was important to analyze perceptions related to the use of various animal identification devices; for example, the local dislike of boluses, paint, and back tags, the preference for small ear tags and cheap devices etc., point to what communities would easily accept and adopt. The data provides guidance on the preferred identification and traceability system, that which is locally acceptable and potentially sustainable.

## Conclusions

We have demonstrated a number of factors that need to be considered for a LIT system to operate in a pastoralist setting. The design requires active participation of all relevant stakeholders in the livestock business. The actors need to be sensitized on the objectives and benefits of developing and utilizing such a system. For example, for farmers, they will need to know why they should tag their animals, and for traders and brokers, they should understand why a traceability system would probe them to define the source and next destination of animals in their possession. Market managers will need to understand why all transactions should be inside the market yards, and for authorities, the need for defining policies that encourage different actors to participate in the design and implementation of the system.

Recommendations generated from the study include the following:

- *Animal identification*: Animals that do not have reliable identification markets by the time they are presented at the primary markets should be tagged by market officials. Ear tags or any other preferred identification device could be purchased by the markets using part of the taxes they collect from producers and traders. In this study, ear tags were highly preferred by market actors and policy makers and could therefore be used as a device of choice. They are also easy to apply, suitable for traceability purposes and their prices have been declining gradually as more companies that manufacture animal identification devices get registered. This strategy will require extensive sensitization of all the market actors for compliance. Market facilities for restraining animals (e.g., during tagging) should also be renovated or constructed and policies that support this practice are enacted.
- *Depth and breadth of the traceability system*: Findings from this study show that it is possible to trace animals back to their villages or epidemiological units or origin even though tagging is done at the primary markets. Sensitization of the market actors about the roles they ought to play in determining the sources of the animals they purchase or sell would ensure that reliable information on the sources of animals tagged is collected. The type of data to be collected could be limited to those required for traceability purposes, i.e., animal identification number, owner or trader identities, and the premises they passed through. If there is need to use the traceability data for other epidemiological analyses, e.g., risk assessment, then additional variables such as those collected under this study would be required (e.g., animal characteristics—age, sex, breed, etc.).
- *Data forms and a database:* This study used electronic forms created using ODK application supported by android operation system in smart phones and an online database hosted at ILRI. Data collected in the markets were therefore available at the end of each day. Paper forms could also be used but they would offer immense challenges since this would require extra personnel for processing the data. Electronic systems are now being used universally and expansion of the mobile network has allowed access to the internet in most towns in eastern Africa. Electronic forms would improve the efficiency of operations and increase the capacity for widening the breadth of the traceability system. Furthermore, in areas where traceability is being used to support security operations, sharing genuine transportation permits online would allow the security agents to verify whether the hard copies presented at the check points are valid.
- *Personnel*: Each market should have at least two people charged with capturing the traceability data. They will need to be trained and issued with protective clothing and masks.
- *Stakeholder feedback:* There should be a process of collating feedback from the stakeholders in order to identify ways of refining the system. This study used a questionnaire survey but other ways of capturing similar data exist including open meetings, setting up special committees, etc.

## Annex 1

**Table 10:** Livestock markets included in the livestock traceability pilot project

Primary markets	Narok (Kenya)–Olposimoru, Oloolaimutia, Lolgorian, Kilgoris markets
	Ngorongoro (Tanzania)–Soit Sambu market
	Cattle were presented for sale at selected primary market outlets by the sellers who could either be farmers or traders. The sellers were briefed about the study – its objectives, procedures and expectation. Consent to participate was subsequently sought. Animals were led into a crush installed next to the market entrance for identification device application. Identification devices used were mainly the ear tags though a few back tags and paint were tested in the course of the study period. Ownership and source details were captured into the database system. On exit from the market (or at the end of the market activities for animals tagged but not sold out) information on who the new owners are as well as the identified animal’s next destination was used to update the system
Secondary markets*	Narosura, Ngoosuani, Ewaso Ng’iro, Chebunyo, Mulot, Ntulele, Suswa markets
	The focus was on animals identified, those already in the database system, and those that passed through specific market routes. On entry, information on ownership, source and next destination was sought. On exit, details of new owners and animal’s next destination were also captured. The process of capturing entry and exit data was repeated every time an animal arrived or departed a specified secondary market until arrival at the terminal markets
Slaughterhouses	Ewaso Ng’iro in Narok and Dagoretti (Nairobi)
	Ownership and source information was captured for identified animals presented for slaughter at these slaughterhouses. Meat inspection results were also captured. Samples (tissue- meat, liver, kidney and blood were collected for residue analysis. Recovered IDs were submitted to the project
